# Association of circulating branched-chain amino acids with cardiometabolic traits differs between adults and the oldest-old

**DOI:** 10.18632/oncotarget.21489

**Published:** 2017-10-04

**Authors:** Liang Sun, Caiyou Hu, Ruiyue Yang, Yuan Lv, Huiping Yuan, Qinghua Liang, Benjin He, Guofang Pang, Menghua Jiang, Jun Dong, Ze Yang

**Affiliations:** ^1^ The MOH Key Laboratory of Geriatrics, Beijing Hospital, National Center of Gerontology, Beijing, China; ^2^ Department of Neurology, JiangBin Hospital, Nanning, Guangxi, China; ^3^ Department of Clinical Laboratory, JiangBin Hospital, Nanning, Guangxi, China; ^4^ College of Life and Environmental Sciences, Hangzhou Normal University, Hangzhou, Zhejiang, China

**Keywords:** BCAAs, cardiometabolic trait, risk factor, age-dependent, the oldest-old

## Abstract

Branched-chain amino acids (BCAAs) are promising for their potential anti-aging effects. However, findings in adults suggest that circulating BCAAs are associated with cardiometabolic risk. Moreover, little information is available about how BCAAs influence clustered cardiometabolic traits in the oldest-old (>85 years), which are the fastest-growing segment of the population in developed countries. Here, we applied a targeted metabolomics approach to measure serum BCAAs in Chinese participants (aged 21-110 years) based on a longevity cohort. The differences of quantitative and dichotomous cardiometabolic traits were compared across BCAAs tertiles. A generalized additive model (GAM) was used to explore the dose-response relationship between BCAAs and the risk of metabolic syndrome (MetS). Overall, BCAAs were correlated with most of the examined cardiometabolic traits. The odds ratios for MetS across the increasing BCAA tertiles were 3.22 (1.70 – 6.12) and 5.27 (2.88 – 9.94, referenced to tertile 1) after adjusting for age and gender (*P*_trend_ < 0.001). The association still existed after further controlling for lifestyle factors and inflammation factors. However, the correlations between circulating BCAAs and quantitative traits were weakened in the oldest-old, except for lipids, the levels of which were distinctly different from those in adults. The stratified analysis also suggested that the risky BCAAs-MetS association was more pronounced in adults than in the oldest-old. Moreover, generalized additive model (GAM)-based curve-fitting suggested that only when BCAAs exceeded a threshold (approximately 450 μmol/L) was the BCAAs-MetS association significant. The relationship might be aging-dependent and was more pronounced in adults than in the oldest-old.

## INTRODUCTION

Cardiometabolic disorders (CMDs) account for more than 30% of all deaths globally and should receive more attention because of the acceleration of aging worldwide [1, 2]. The oldest-old, *i.e*., those age 85 and older, are the fastest-growing segment of the U.S. population. Moreover, the evaluation of the nutritional status of the populatio and its association with CMD-related metabolic profiles has been considered a key component in comprehensive geriatric assessments (CGAs), which have been proven to be helpful for survival and for relieving the late-life burden of frailty [3]. Metabolic syndrome (MetS) and its components play important roles in the origin of CMDs [4]. The development of metabolomics has enabled the study of the role of small-molecule nutrients in CMD-related metabolic phenotypes [5]. In contrast to genomic markers, metabolites are relatively dynamic and represent cellular activity and the effects of extrinsic exposures.

Branched-chain amino acids (BCAAs), namely, leucine, isoleucine, and valine, are a cluster of essential amino acids that cannot be synthesized *de novo*. BCAAs play crucial roles in development and aging [6]. Circulating BCAAs are directly taken up by skeletal muscle and used for energy, muscle repair, or building. Thus, BCAAs are essential in the maintenance of muscle content and anabolic effects because they improve protein synthesis, decrease the rate of protein degradation [7], and result in a positive net energy balance. BCAAs were first found to prolong chronological life in yeast and were thereafter marked as potential candidates for promoting survival [8]. Then, in male mice, an original BCAA mixture was proven to extend the average life span, and the mechanism was related to increased mitochondrial biogenesis and reduced oxidative stress in cardiac and skeletal muscles via endothelial nitric oxide synthase-mediated mechanisms [9].

Despite the beneficial anti-aging findings in animal models, most observational studies in humans have suggested the opposite effect. Based on primarily middle-aged and young-elderly subjects, circulating BCAAs have been reported to be risk factors for obesity, diabetes and coronary artery disease [10–13], and these findings have even dramatically led the public to consider BCAAs as adverse ingredients. Human aging is characteristically accompanied by a progressive loss of skeletal muscle mass, *i.e.,* sarcopenia, which might be attributable to a potential age-related stratification of muscle anabolism and metabolic balance [14]. Moreover, because skeletal muscle is at the core of metabolic homeostasis, older subjects might possess a distinct metabolic status due to frailty, with associated muscle mass decline compared to adults. However, because few subjects aged over 85 years have been included in the existing observational studies about the relationship between BCAAs and cardiometabolic traits, little is known about how circulating BCAAs influence these factors in the oldest-old.

To fill this gap in knowledge, we applied a targeted metabolomics approach to determine the whole-lifespan profile of circulating BCAA levels with chronological ages based on a subset of longitudinal longevity cohort covering a wide age range (21 to 110 years). Further, we assessed the association of BCAAs with MetS and its components while considering the confounding factors of aging stage and gender.

## RESULTS

Of the 611 included participants, 317 (51.9%) were men, and 109 (17.8%) displayed symptoms consistent with the diagnosis of MetS (Table 1). The average (standard deviation, SD) concentrations of total BCAAs, valine, leucine, and isoleucine were 432.0 (95.8), 236.8 (50.7), 125.8 (30.4), and 69.3 (18.0) μmol/L, respectively. Subjects with higher BCAA levels were more likely to be younger and male. Additionally, with increasing circulating BCAAs, subjects tended to have elevated body mass index (BMI), fasting plasma glucose (FPG), low-density lipoprotein cholesterol (LDL-C), triglycerides (TG), and a greater number of accumulated cardiometabolic dichotomous traits, with the exception of reduced high-density lipoprotein cholesterol (HDL-C) (all adjusted *P*_trend_ <0.05).

**Table 1:** Characteristics of study participants by serum BCAA Tertiles

Characteristic^a^	Tertile of serum BCAA			*P*_trend_^b^
	Low < 393.1 umol/L	Middle 393.1 - 468.2 umol/L	High >468.2 umol/L	
Number	204	203	204	
Age, years	75.3 ± 23.1	64.5 ± 20.2	62.6 ± 20.6	<0.001
Gender, male%	34.8	52.7	68.1	<0.001
BCAA, umol/L	330.7 ± 49.1	430.4 ± 20.7	534.8 ± 61.6	<0.001
Valine, umol/L	183.9 ± 27.1	236.8 ± 14.9	289.6 ± 34.1	<0.001
Isoleucine, umol/L	52.3 ± 8.9	67.9 ± 7.5	87.7 ± 14.4	<0.001
Leucine, umol/L	94.5 ± 17.7	125.6 ± 9.3	157.4 ± 19.6	<0.001
BMI, kg/m^2^	20.6 ± 3.5	22.8 ± 3.7	23.8 ± 3.6	<0.001
FPG, mmol/L	5.8 ± 1.5	5.7 ± 1.0	6.3 ± 1.5	0.017
HBA1c, %	5.2 ± 0.4	5.3 ± 0.4	5.4 ± 0.7	0.417
C-peptide, ng/ml	4.5 (1.0-15.7)	4.4 (1.1-20.2)	5.8 (1.5-19.9)	0.001
SBP, mmHg	132.9 ± 20.9	133.2 ± 21.2	133.1 ± 19.2	0.923
DBP, mmHg	76.6 ± 11.7	77.6 ± 9.8	78.4 ± 9.9	0.082
HDL-C, mmol/L	1.43 ± 0.40	1.37 ± 0.30	1.26 ± 0.25	<0.001
LDL-C, mmol/L	2.75 ± 0.87	2.99 ± 0.82	2.97 ± 0.87	0.010
TC, mmol/L	4.8 ± 1.0	5.0 ± 0.9	4.9 ± 0.9	0.060
TG, mmol/L	1.3 ± 1.1	1.6 ± 1.1	2.0 ± 1.5	<0.001
hsCRP, mg/L	3.0 ± 3.5	1.9 ± 2.8	2.0 ± 2.6	0.064
IL6, pg/mL	8.3 ± 11.2	5.6 ± 3.8	8.2 ± 6.4	0.573
Number of Cardiometabolic dichotomous traits (%) ^c^				<0.001
0	67 (32.8)	47 (23.1)	40 (19.6)	-
1	77 (37.7)	71 (34.9)	48 (23.5)	-
2	44 (21.5)	47 (23.1)	61 (29.9)	-
3	14 ( 6.8)	28 (13.7)	34 (16.6)	-
4	2 ( 0.9)	9 ( 4.4)	16 ( 7.8)	-
5	0 ( 0.0)	1 ( 0.5)	5 ( 2.5)	-
Metabolic syndrome, n (%) ^c^	16 (7.8)	38 (18.7)	55 (26.9)	<0.001

Abbreviations: BCAA, branched-chain amino acid; BMI, body mass index; SBP, Systolic blood pressure; DBP, Diastolic blood pressure; FPG, fasting plasma glucose; TC-total cholesterol; TG- triglyceride; HDL-C, high density lipoprotein cholesterol; LDL-C, low density lipoprotein cholesterol.

^a^ Data are mean ± SD, median (interquartile range) for continuous variables, or percentage for categorical variables.

^b^ *P* values for the overall comparisons across BCAA Tertiles adjusting for age, gender, smoke and alcohol drinking, except for itself.

^c^ Defined according to the criteria of metabolic syndrome from Chinese Diabetes Society, CDS ( 2004) for Chinese.

**Figure 1:**
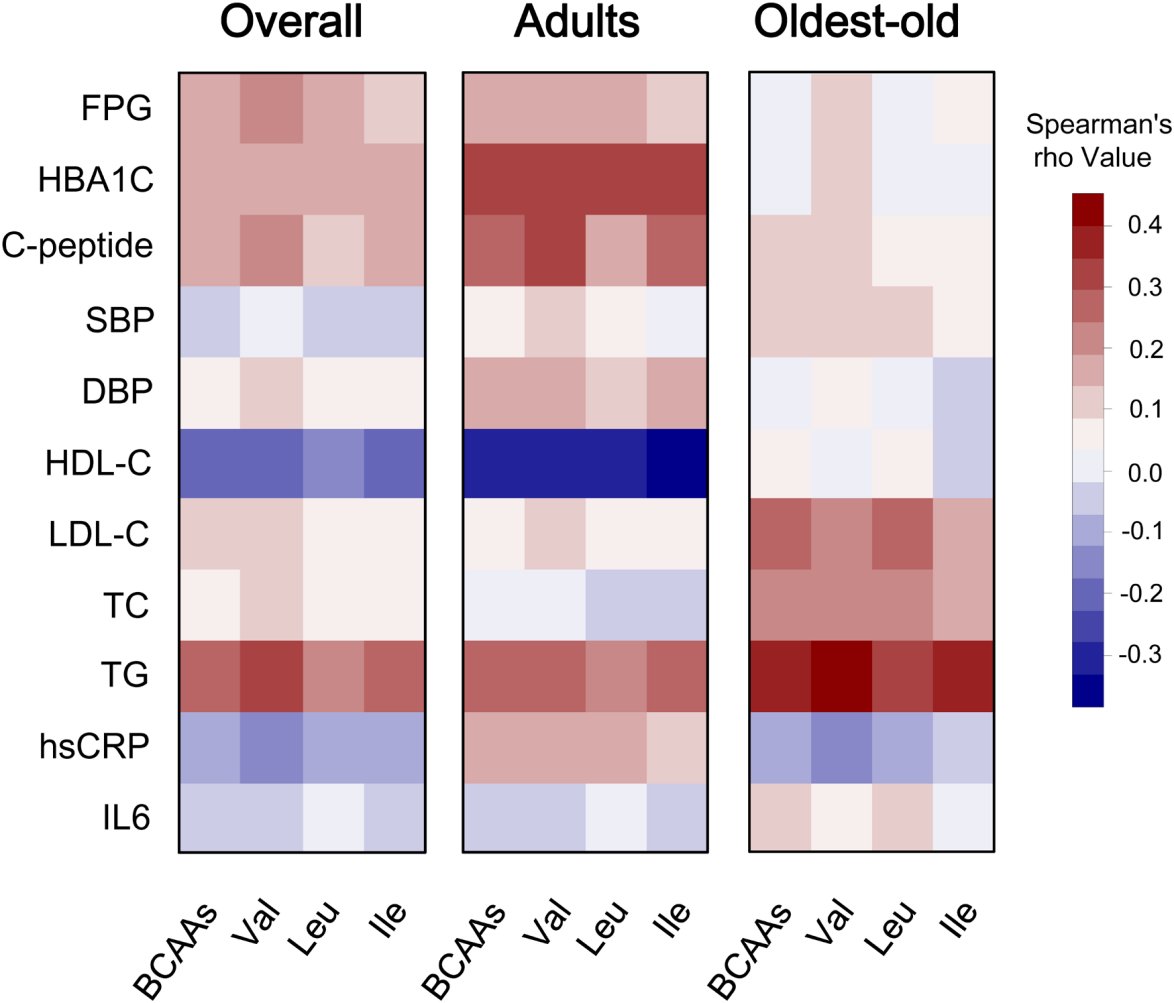
Correlation heatmap illustrating the relationship between circulating BCAAs and cardiometabolic quantitative phenotypes Spearman correlation coefficients are presented in a blue-white-red color scheme. Dark red indicates a more positive correlation, and dark blue indicates a more negative correlation; white indicates no correlation. We present the corresponding heatmaps for the overall cohort, the adults and the oldest-old subjects.

**Figure 2:**
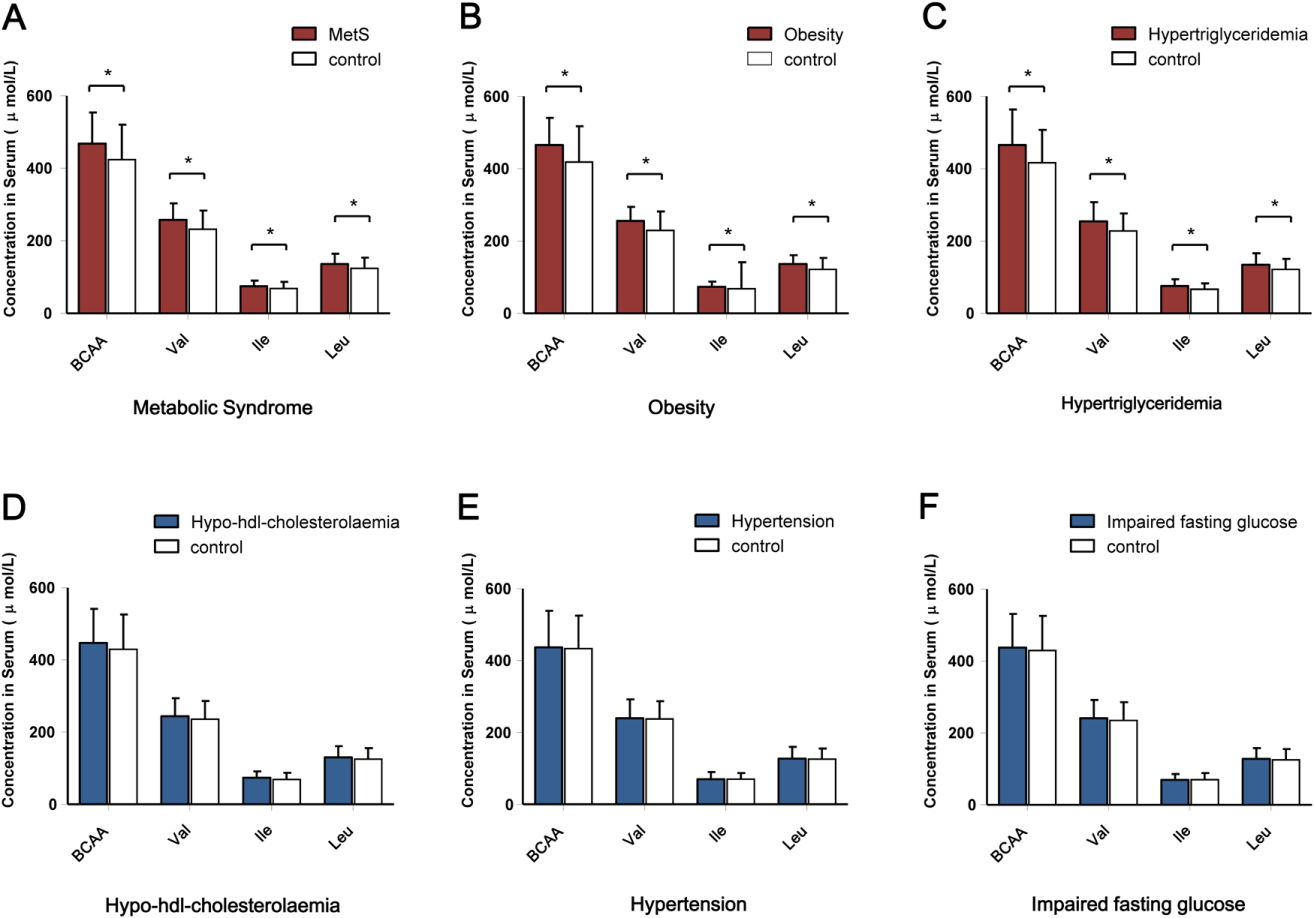
Comparison of the circulating BCAA levels according to the dichotomous cardiometabolic traits **(A)** Serum BCAA levels according to the presence of MetS. **(B)** Serum BCAAs levels according to the presence of obesity. **(C)** Serum BCAA levels according to the presence of hypertriglyceridemia. **(D)** Serum BCAA levels according to the presence of hypo-HDL- cholesterolemia. **(E)** Serum BCAA levels according to the presence of hypertension. **(F)** Serum BCAA levels according to the presence of impaired fasting glucose. ^*^P < 0.001.

The correlation between BCAAs (both individual components and overall amino acids) and quantitative cardiometabolic phenotypes were analyzed. Overall, BCAAs were positively correlated with fasting glucose, C-peptide and TG and were negatively correlated with HDL-C. However, the stratified analysis by aging stage revealed an obviously age-dependent profile between the adults and the oldest-old subgroup. Compared with the adults, the oldest-old exhibited a distinct pattern in the correlations between circulating BCAAs and most quantitative traits. Specifically, the oldest-old showed weakened correlations related to glycemic traits, blood pressure, and HDL-C and increased correlations related to blood lipids compared with the adults. Regarding the inflammation markers, the adults and oldest-old even presented an opposite weak correlation with BCAAs levels (Figure 1). The further stratified analysis by gender also revealed that a potential gender-specific difference existed in both adults and the oldest-old subjects (Supplementary Figure 1). Regarding the patterns of dichotomous cardiometabolic traits, circulating BCAAs were found to be higher in subjects with MetS, obesity and hypertriglyceridemia compared with the corresponding control groups (*P* < 0.001; Figure 2A-2C). No differences existed between the BCAAs and the other three dichotomous traits, namely, hypo-HDL-cholesterolemia, hypertension and impaired fasting glucose (IFG) (Figure 2D-2F).

Taking the subjects in the lowest BCAA tertile as the reference group, the odds ratios (ORs) for MetS, obesity and hypertriglyceridemia presented an increasing trend across the middle and high tertiles (*P*_trend_ < 0.001) (model 1). After adjusting for age, gender (model 2) and additionally controlling for smoking status, alcohol drinking, tea drinking, high-sensitivity C-reactive protein (hsCRP) and interleukin-6 (IL6), associations of BCAAs with MetS and hypertriglyceridemia were also present (*P*_trend_ < 0.05; model 3) (Table 2).

**Table 2:** Odds ratios (95% Confidence intervals) for MetS, obesity and hypertriglyceridemia according to tertiles of BCAAs concentrations

		Tertile of serum BCAAs			*P*_trend_^a^
		Low < 393.1 umol/L	Middle 393.1 - 468.2 umol/L	High >468.2 umol/L	
MetS	Cases/ total	16/ 204	38/ 203	55/ 204	
	Model 1 ^b^	1	2.71 (1.46, 5.03)	4.34 (2.39, 7.88)	<0.001
	Model 2 ^c^	1	3.22 (1.70, 6.12)	5.27 (2.80, 9.94)	<0.001
	Model 3 ^d^	1	3.93 (0.72, 21.49)	6.71 (1.33, 33.92)	0.017
obesity	Cases/ total	25/ 204	69/ 203	83/ 204	
	Model 1 ^b^	1	3.67 (2.20, 6.11)	4.90 (2.96, 8.11)	<0.001
	Model 2 ^c^	1	3.70 (2.18, 6.28)	4.64 (2.73, 7.89)	<0.001
	Model 3 ^d^	1	2.41 (0.66, 8.78)	2.99 (0.85, 10.51)	0.094
hypertriglyceridemia	Cases/ total	35/ 204	71/ 203	87/ 204	
	Model 1 ^b^	1	2.60 (1.63, 4.13)	3.59 (2.27, 5.67)	<0.001
	Model 2 ^c^	1	2.96 (1.83, 4.78)	4.41 (2.69, 7.20)	<0.001
	Model 3 ^d^	1	4.46 (1.92, 10.37)	6.11 (2.66, 14.03)	<0.001

^a^ *P* values for the overall comparisons across BCAAs Tertiles.

^b^ Model 1: crude risk for MetS.

^c^ Model 2: adjusted for age and gender.

^d^ Model 3: additionally adjusted for smoking status, alcohol drinking , tea drinking, hsCRP and IL6.

**Figure 3:**
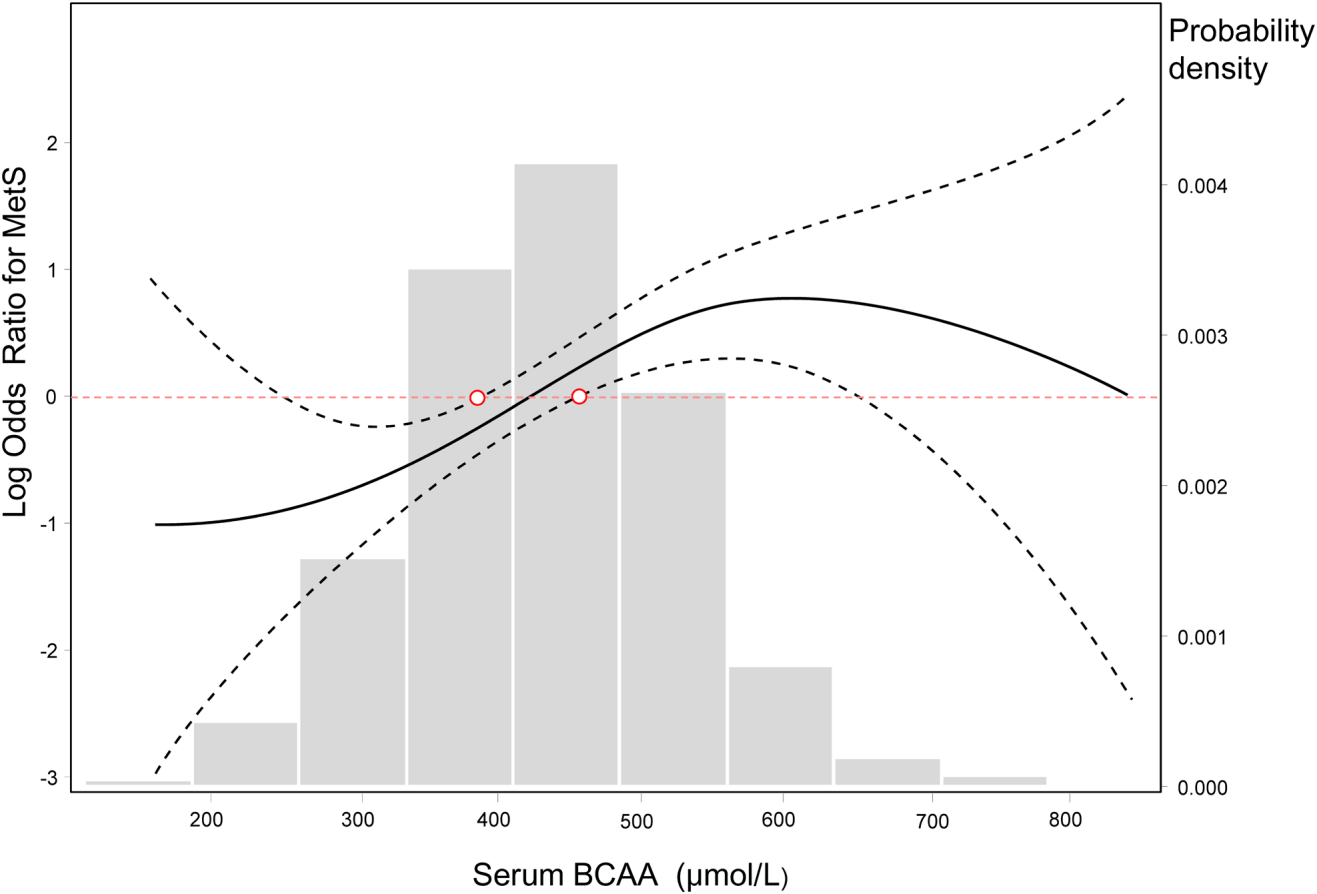
Log-transformed odds ratios for MetS by circulating BCAA levels The lines represent the log-transformed odds ratios (95%CI) based on a generalized additive model (GAM) for BCAA levels. The cutoff point of the log ORs is 0, which corresponds to 1 on the ORs cutoff point. The bars represent the probability density, and 10 equally sized bins were set. *P*_smooth terms_ <0.001, and the estimated degrees of freedom for the model terms, namely, *edf* = 2.646.

When the BCAA concentration was considered as a continuous variable, based on a generalized additive model, we fit a smooth curve that presented the dose-response relationship between BCAAs and the log-ORs for MetS (*P*_smooth terms_ <0.001, *edf* = 2.646), which suggested a cut-off threshold existed and corresponded to the intersection of the red horizontal line of logORs = 0 and the lower 95% confidence intervals (CIs) curve. This threshold decided whether BCAAs were associated with the risk of MetS (Figure 3). BCAAs could be considered risk biomarkers for MetS only when the serum concentration exceeded this threshold (approximately 450 μmol/L). Additionally, histogram suggested a plausible normal distribution in our study participants.

**Figure 4:**
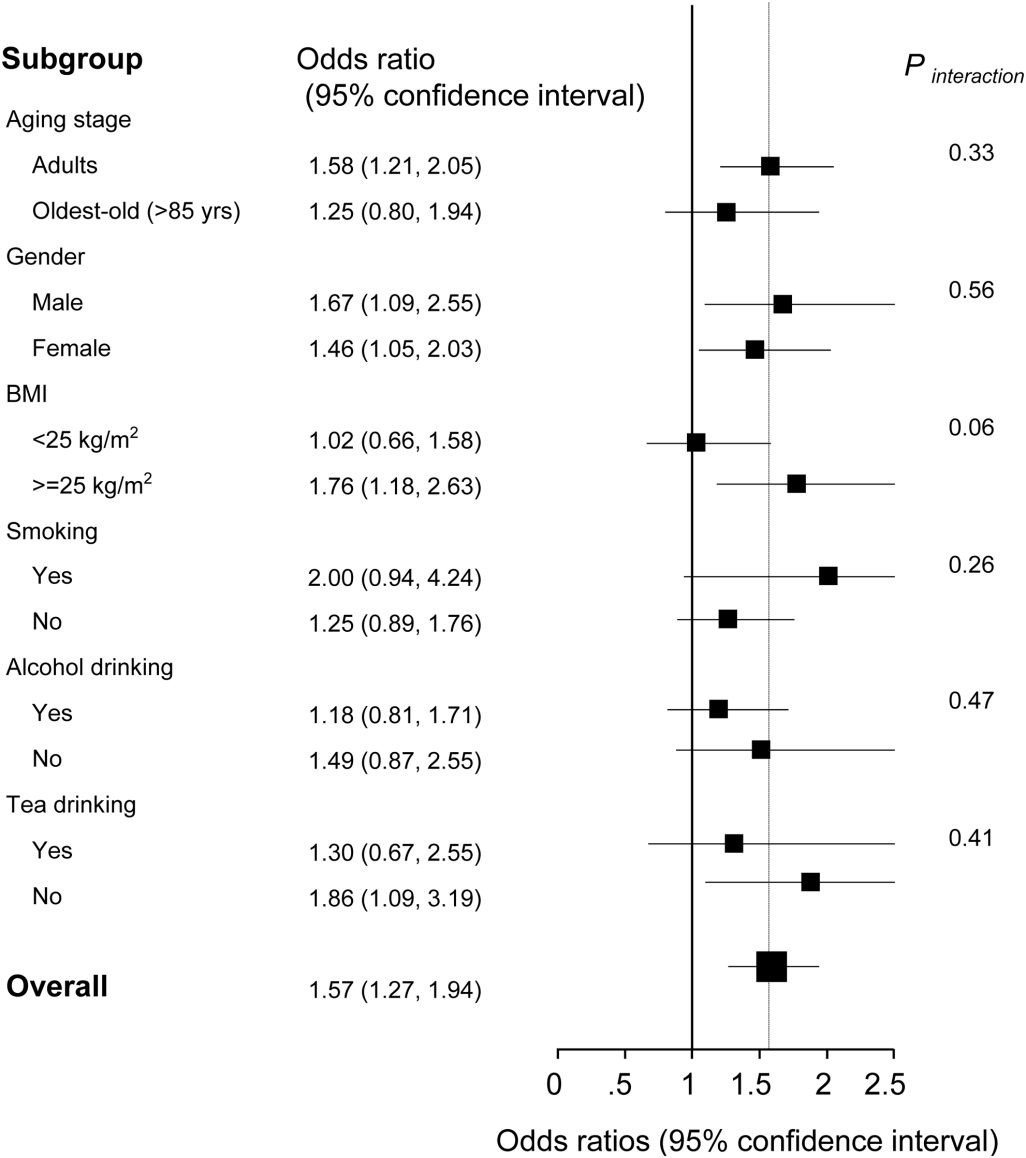
Stratified analysis of the association [odds ratios (95% confidence intervals)] between circulating BCAA levels (per 1-SD increment) and MetS ^a^ Adjusted for age, gender, smoking status, alcohol drinking, tea drinking, C-reactive protein and interleukin-6, stratifying factors excepted.

Although the overall OR for MetS was 1.57 (1.27-1.94) per 1-SD increment of BCAAs in the multivariable adjusted models, the stratified analysis suggested the BCAAs-MetS association was more pronounced in adults, obese individuals, current smokers, and participants with no habit of drinking tea compared with their counterparts (Figure 4). In the oldest-old and non-obese subjects, the risky BCAAs-MetS association was not observed. No interaction was detected (all *P*_interaction_ > 0.05).

**Figure 5:**
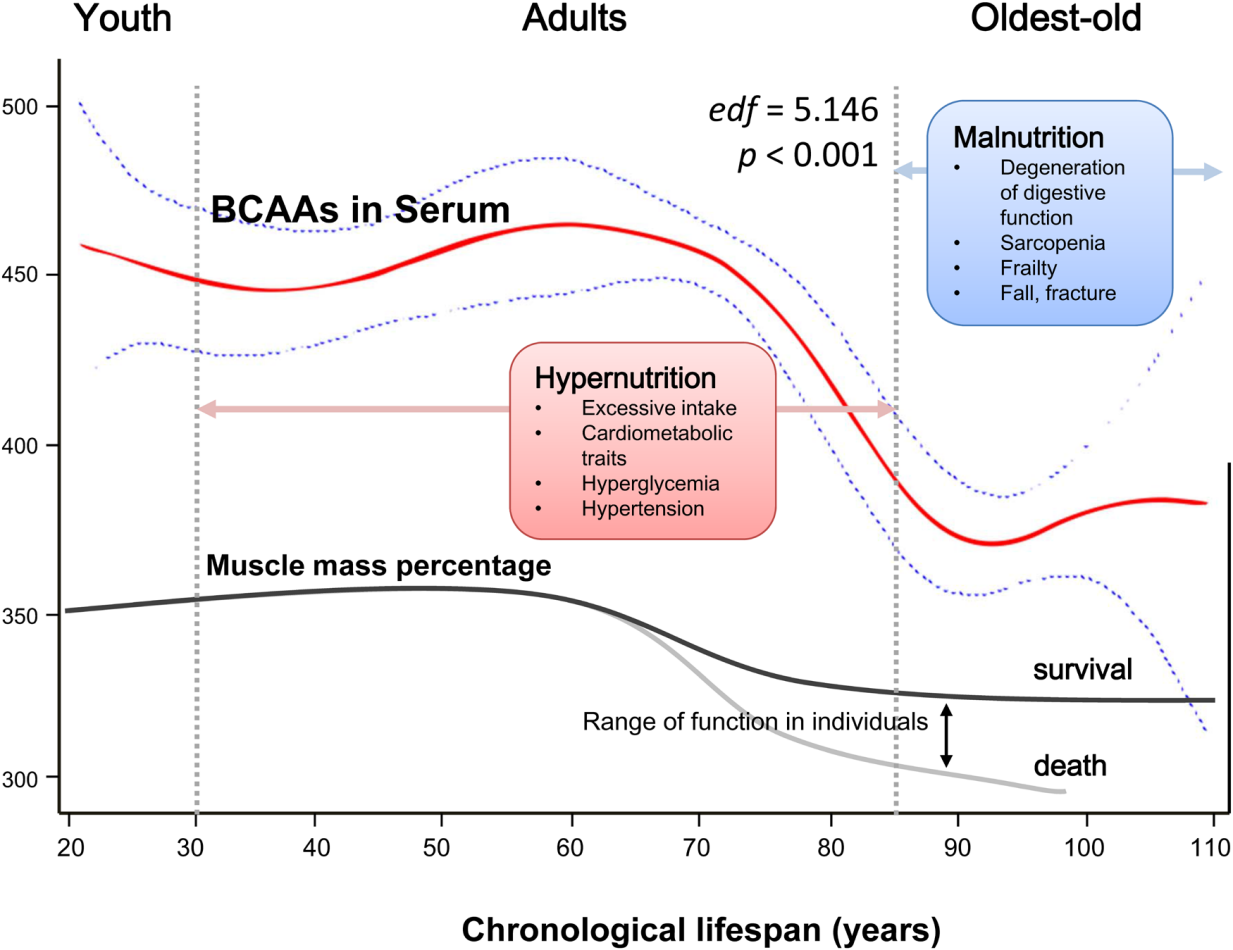
Schematic of a potential hypothesis for the whole-lifespan profile of circulating BCAA levels A generalized additive model (GAM) was applied to fit a smooth curve according to our participants (21 to 110 years old). *P*_smooth terms_ <0.001, and the estimated degrees of freedom for the model terms, namely, *edf* = 5.146. As a nutrient-related metabolomics biomarker, BCAAs might link more to malnutrition in the oldest-old subjects due to factors such as sarcopenia and frailty rather than hypernutrition-related cardiometabolic diseases in adults.

Finally, based on the GAM, we fit a smooth regression curve that represented a continuous relationship between chronological age (21 to 110 years) and serum BCAAs (*P*_smooth terms_ <0.001, *edf* = 5.146; Figure 5). A plausible dynamic pattern of circulating BCAA levels with human aging was indicated. Before reaching 60 years of age, circulating BCAAs were stable at a high level (at approximately 450 μmol/L), and in the next 25 years, the level declined rapidly until 90 years old, when the BCAAs became stable at a low level (at approximately 370 μmol/L), which was highly consistent with the trend of aging-related muscle mass decline.

## DISCUSSION

Recently, BCAAs have come under the academic spotlight as essential metabolic intermediates and nutrient signals. However, the reputation of BCAAs is still paradoxical [6, 9, 10, 15–18]. Some studies suggest BCAAs might improve metabolism and simultaneously prolong lifespan, while other studies suggest that BCAAs are metabolic risk biomarkers or even the immediate cause of deterioration in insulin sensitivity.

Although the pace of global aging is increasing rapidly, most of the medical guidelines for elderly subjects are still derived from studies of adults. In the present study, based on a cohort with a wide age range (21 to 110 years), we studied the relationship between circulating BCAAs and cardiometabolic traits, and we were the first to find that this relationship is age-dependent. Moreover, we examined the relationship between BCAA concentrations and chronological age across whole-lifespan and found that BCAAs can act as risk biomarkers for most quantitative and dichotomous cardiometabolic traits, such as MetS, only when their concentrations exceeded a certain threshold. These findings are of particular significance because they suggest that BCAAs might not be risk biomarkers of cardiometabolic traits in elderly subjects who suffer from different levels of malnutrition.

In general, we identified a positive correlation between BCAAs and cardiometabolic traits. According to the tertiles of BCAA levels, we found that BMI, FPG, LDL-C, TG and the accumulated number of cardiometabolic dichotomous traits increased with increases in BCAAs (Table 1). Many studies in humans and rodent models support the view that circulating BCAAs are associated with obesity and insulin resistance metabolic disorders [10, 19–23]. However, BCAAs have been considered to be beneficial for the regulation of body weight, muscle protein synthesis, glucose homeostasis, lipid metabolism, and the aging process in other studies [16, 17, 24]. This inconsistency is difficult to understand.

The reason and mechanism underlying these conflicting findings should be carefully considered, and there are at least two areas of improvement. Firstly, cohorts with wide age range are needed, especially cohorts that include the elderly and oldest-old, which have rarely been included but should be valued because of the acceleration of aging worldwide. It has been reported that BCAAs can trigger different and even opposite effects, depending on the catabolic or anabolic states of the organisms [25]. Indeed, human aging is accompanied by a potential shift from an anabolic state (early and midlife) to a catabolic state (late life). Secondly, there is lack of both reference measurement procedures and reference ranges for BCAAs. Therefore, it is difficult to compare data from different studies because of their non-standardized origins. Moreover, to define a biomarker, we should know its dose-response relationship and thresholds. Additionally, it is also necessary to understand the baseline dynamic profile of circulating BCAAs with human aging to precisely assess the role of BCAAs at different ages.

We conducted an analysis that was stratified by aging stage and revealed a distinct aging-dependent correlation between circulating BCAAs and quantitative cardiometabolic phenotypes (Figure 1). Generally, BCAAs were associated with cardiometabolic phenotypes more strongly in the adult subgroup; however, regarding the older subjects, the correlations weakened. These findings are consistent with our previous hypothesis that aging might be accompanied by a potential shift from an anabolic state (early and midlife) to a catabolic state (late life), which might have been neglected in most previous studies that included a few oldest-old participants aged over 85 years.

In the analysis of the dichotomous cardiometabolic traits, we found that circulating BCAAs were significantly higher in the participants with MetS, obesity and hypertriglyceridemia relative to their counterparts (Figure 2A-2C). These three traits, especially hypertriglyceridemia, are all closely linked to high-fat diets and alcohol use [26]. However, regarding the other dichotomous traits, which are not directly influenced by high-fat diets, no differences were observed (Figure 2D-2F). We fit a smooth curve that presented the dose-response relationship between BCAAs and the log-ORs for MetS and suggested that BCAAs were associated with MetS only when the BCAA levels exceeded a threshold (approximately 450 μmol/L; Figure 3). To some extent, the threshold cut-off might simply reflect a switching from an anabolic state to a catabolic state. The multivariable adjusted stratified analysis suggested the BCAAs-MetS association was more pronounced in middle-aged adults, obese individuals, current smokers, and participants with no habit of drinking tea compared with their counterparts (Figure 4). We should also notice potential confounding effects from lifestyle factors. At least, smoking is risky and tea drinking is protective for the association between BCAA and MetS. Moreover, from the lifespan perspective, we found that the BCAA level and chronological age were highly synchronized (Figure 5). In the adult phase, BCAAs were stable at a high level with an ideal muscle mass percentage, and the subjects were partly in a state of hypernutrition. Within the next 25 years, the circulating BCAAs declined rapidly. This decline was accompanied by a reduction of muscle mass, and the subjects switched to a state of malnutrition. It has been reported that whole body protein synthesis in humans (g/kg body weight/ day) drastically decreases with age, being 3.0 in adults, and 1.9 in elderly subjects [27]. Because aging and systematic degeneration might have a potential influence, and the findings in adults and older subjects are difficult to compare, it is necessary to evaluate the role of circulating BCAAs according to aging stage.

We believe that circulating BCAAs *per se* might not causally increase the risk of insulin resistance. Firstly, although BCAAs can activate mTORC1, they are not necessary or sufficient to trigger insulin resistance [28]. Secondly, insulin resistance or metabolic deficits can decrease BCAA catabolism [12]. Therefore, in the adult stage, circulating BCAAs are more likely to be a consequence of, and act as markers of, the loss of insulin action. Thirdly, in addition to s catabolism, circulating BCAAs are also influenced by a BCAA-containing diet. Other ingredients contained in the diet, such as lipids, might confound the real relationship between BCAAs and cardiometabolic traits in subjects who are more exposed to high-fat diets, such as men and smokers compared with women and non-smokers. Fourth, in the older subjects experiencing decline in both their digestive system and their dental chewing ability compared with adults, the real role of BCAAs might be detected because they are physiologically removed from the diet-related confounds. Furthermore, dietary supplementation with BCAAs has been reported to elicit anti-aging effects [29]. Even for elderly diabetes patients, a long-term randomized study suggested that BCAA supplementation could improve metabolic control [30]. Therefore, the findings regarding circulating BCAAs in middle-aged subjects might more likely reflect potential confounding risk factors related to food, such as fat and carbohydrates, unless the subjects are extremely limited in terms of dietary components.

Based on a cohort with a wide age range, the present study is the first to examine the relationship between BCAAs and a set of cardiometabolic traits and found that this relationship is age-dependent. Only when people were in the adult stage and their circulating BCAAs were above a certain threshold of concentration could BCAAs act as a risk biomarker for most of the cardiometabolic traits. Several limitations should be considered in the future. First, due to the cross-sectional design of the study, we were unable to establish causality. As this study was based on a subset of our ongoing longevity cohort, we will dedicate prospective study on this issue in the future. Secondly, due to a lack of data about the diets and calories of our subjects, we did not perform the corresponding analyses. Thirdly, how body composition is involved in circulating BCAAs, cardiometabolic traits and human aging remains unknown.

Our study demonstrated for the first time that increased circulating BCAAs are associated with most cardiometabolic traits, including MetS, in a Chinese cohort with a wide age range (21-110 years). The relationship might be age-dependent and was obviously more pronounced in adults than in the oldest-old. From the perspective of gerontology, these findings highlight the importance of nutrients as biomarkers that should be included in the CGAs in old age, which is a distinct physiological stage from adulthood.

## MATERIALS AND METHODS

### Study participants

The current study was a cross-sectional study based on a subset of the Longevity and Health of Aging Population in Guangxi China (LHAPGC) Cohort in Yongfu County [31], which was qualified as a Chinese Longevity Town by the Geriatric Society of China in 2007. The participants were recruited between 2008-2016 by a quota and judgment sampling method that covered nearly all the oldest-old and young control subjects in each village of Yongfu County. According to the sufficiency of the remaining volume of serum for mass spectrometry assays, 611 eligible subjects aged 21 to 110 years were included in the present study (317 men and 294 women). All of the participants self-reported as being of Han nationality.

This study was conducted according to the guidelines laid out in the Declaration of Helsinki and all procedures involving human subjects were approved by the Ethics Committee of Beijing Hospital. All participants or their guardians provided written informed consent.

### Data collection

Trained and qualified personnel conducted the household visits. Information about demographic variables, health status, current smoking (yes or no), alcohol drinking (yes or no) and tea drinking (yes or no) were obtained using a standardized questionnaire. The peripheral fasting serum samples were separated for laboratory testing of the lipid and glucose parameters using previously described methods [32]. Serum samples were isolated and stored at -80°C until analysis.

### Serum BCAAs measurement

As previously reported, a targeted metabolomics approach based on isotope dilution liquid chromatography tandem mass spectrometry (LC/MS/MS) method was adopted for BCAAs measurement [21, 33]. Briefly, 0.05 mL aliquots of calibrators or serum samples were mixed with 0.05 mL of the isotopically labeled internal standard solution. The amino acids were extracted with 0.4 mL of acetonitrile containing 0.1% formic acid and analyzed using LC/MS/MS with positive electronic spray ionization in the multiple-reaction monitoring mode. MS/MS was carried out on an API4000 triple quadruple mass spectrometer (Applied Biosystems, USA).

### Cardiometabolic traits

We studied the following quantitative cardiometabolic phenotypes: 1) glycemic traits including FPG, hemoglobin A1c (HBA1c), and fasting C-peptide; 2) diastolic and systolic blood pressure; 3) lipid metabolism including HDL-C, LDL-C, total cholesterol (TC), and TG; and 4) inflammation markers including hsCRP and IL6. Prior to the analysis, the variable fasting C-peptide was transformed to the natural logarithmic scale.

Additionally, we studied six dichotomous cardiometabolic traits, including MetS, obesity, hypertriglyceridemia, hypo-HDL-cholesterolemia, IFG and hypertension. The diagnoses of MetS and its components were made according to the criteria provided by the Chinese Diabetes Society (2004) [34].

### Statistical methods

Continuous variables of normal distribution were presented as mean ±SD; non-normal variables were presented as median (interquartile range). For continuous variables, covariance analysis was conducted to compute the difference across the BCAA tertiles, and for the categorical variables, multivariate logistic regression was conducted to compute the differences across the BCAA tertiles. When appropriate, log10 transformations of skewed variables were used in the analyses.

We conducted non-parametric Spearman correlation analyses to describe the correlations between circulating BCAA levels and cardiometabolic quantitative phenotypes.

The unconditional multivariate logistic regression model was used to estimate the ORs and their 95% CIs for binary outcomes, such as MetS, for each BCAA tertile compared with the lowest tertile with adjustments for age (continuous), gender, current smoking status (yes, no), alcohol drinking (yes, no), and tea drinking (yes, no).

We applied a GAM, which replaced the linear form $\sum \beta_{j}X_{j}$ with a sum of smooth functions $\sum S_{j}\left(X_{j}\right)$ to fit a smooth curve for a continuous dose-response relationship between circulating BCAA level and MetS risk, with an increment of per unit BCAAs. The GAM uses a link function to establish a relationship between the mean of the response variable and a “smoothed” function of the explanatory variable(s). These models are useful when the relationship between variables is expected to be complex and not easily fit to standard linear or non-linear models [35].

Stratified analyses were performed according to aging stage (adults: age <75 years and oldest-old: age >85 years), gender, BMI category (<25, ≥25), current smoking status, alcohol drinking, and tea drinking. The ORs (95% CIs) were calculated in logistic models with a 1-SD increment used for the odds ratio calculations. In addition, likelihood ratio tests were conducted to examine interactions.

Moreover, a GAM was also used to find a plausible smooth curve for a continuous relationship between circulating BCAA level and chronological lifespan (21 to 110 years) to find clues about the dynamics of BCAAs in relation to human aging.

Two-sided *P* values < 0.05 were considered statistically significant. All statistical analyses were performed with STATA 10.0 software (StataCorp, College Station, TX, USA) and the statistics package R version 3.0.2 (R Development Core Team 2013).

## SUPPLEMENTARY MATERIALS TABLE



## Abbreviations

BCAAs: branched-chain amino acids
BMI: body mass index
CGAs: comprehensive geriatric assessments
CIs: confidence intervals
CMDs: cardiometabolic disorders
FPG: fasting plasma glucose
GAM: generalized additive model
HBA1c: hemoglobin A1c
HDL-C: high-density lipoprotein cholesterol
hsCRP: high-sensitivity C-reactive protein
IFG: impaired fasting glucose
IL6: interleukin-6
LC/MS/MS: liquid chromatography tandem mass spectrometry
LDL-C: low-density lipoprotein cholesterol
LHAPGC: Longevity and Health of Aging Population in Guangxi China
MetS: metabolic syndrome
ORs: odds ratios
SD: standard deviation
TC: total cholesterol
TG: triglycerides

## References

[1] Wang H, Dwyer-Lindgren L, Lofgren KT, Rajaratnam JK, Marcus JR, Levin-Rector A, Levitz CE, Lopez AD, Murray CJ (2012). Age-specific and sex-specific mortality in 187 countries, 1970-2010: a systematic analysis for the Global Burden of Disease Study 2010. Lancet.

[2] Lunenfeld B, Stratton P (2013). The clinical consequences of an ageing world and preventive strategies. Best Pract Res Clin Obstet Gynaecol.

[3] Tortelli R, Lozupone M, Guerra V, Barulli MR, Imbimbo BP, Capozzo R, Grasso A, Tursi M, Di Dio C, Sardone R, Giannelli G, Seripa D, Misciagna G (2017). Midlife metabolic profile and the risk of late-life cognitive decline. J Alzheimers Dis.

[4] Ford ES (2005). Risks for all-cause mortality, cardiovascular disease, and diabetes associated with the metabolic syndrome: a summary of the evidence. Diabetes Care.

[5] Cheng S, Shah SH, Corwin EJ, Fiehn O, Fitzgerald RL, Gerszten RE, Illig T, Rhee EP, Srinivas PR, Wang TJ, Jain M (2017). Potential impact and study considerations of metabolomics in cardiovascular health and disease: a scientific statement from the American Heart Association. Circ Cardiovasc Genet.

[6] Newgard CB, An J, Bain JR, Muehlbauer MJ, Stevens RD, Lien LF, Haqq AM, Shah SH, Arlotto M, Slentz CA, Rochon J, Gallup D, Ilkayeva O (2009). A branched-chain amino acid-related metabolic signature that differentiates obese and lean humans and contributes to insulin resistance. Cell Metab.

[7] Kimball SR, Jefferson LS (2006). Signaling pathways and molecular mechanisms through which branched-chain amino acids mediate translational control of protein synthesis. J Nutr.

[8] Alvers AL, Fishwick LK, Wood MS, Hu D, Chung HS, Dunn WA, Aris JP (2009). Autophagy and amino acid homeostasis are required for chronological longevity in Saccharomyces cerevisiae. Aging Cell.

[9] D'Antona G, Ragni M, Cardile A, Tedesco L, Dossena M, Bruttini F, Caliaro F, Corsetti G, Bottinelli R, Carruba MO, Valerio A, Nisoli E (2010). Branched-chain amino acid supplementation promotes survival and supports cardiac and skeletal muscle mitochondrial biogenesis in middle-aged mice. Cell Metab.

[10] Wang TJ, Larson MG, Vasan RS, Cheng S, Rhee EP, McCabe E, Lewis GD, Fox CS, Jacques PF, Fernandez C, O'Donnell CJ, Carr SA, Mootha VK (2011). Metabolite profiles and the risk of developing diabetes. Nat Med.

[11] Rauschert S, Uhl O, Koletzko B, Hellmuth C (2014). Metabolomic biomarkers for obesity in humans: a short review. Ann Nutr Metab.

[12] Batch BC, Hyland K, Svetkey LP (2014). Branch chain amino acids: biomarkers of health and disease. Curr Opin Clin Nutr Metab Care.

[13] Patel MJ, Batch BC, Svetkey LP, Bain JR, Turer CB, Haynes C, Muehlbauer MJ, Stevens RD, Newgard CB, Shah SH (2013). Race and sex differences in small-molecule metabolites and metabolic hormones in overweight and obese adults. OMICS.

[14] Dardevet D, Rieu I, Fafournoux P, Sornet C, Combaret L, Bruhat A, Mordier S, Mosoni L, Grizard J (2003). Leucine: a key amino acid in ageing-associated sarcopenia?. Nutr Res Rev.

[15] Eller LK, Saha DC, Shearer J, Reimer RA (2013). Dietary leucine improves whole-body insulin sensitivity independent of body fat in diet-induced obese Sprague-Dawley rats. J Nutr Biochem.

[16] Nagata C, Nakamura K, Wada K, Tsuji M, Tamai Y, Kawachi T (2013). Branched-chain amino acid intake and the risk of diabetes in a Japanese community: the Takayama study. Am J Epidemiol.

[17] Valerio A, D'Antona G, Nisoli E (2011). Branched-chain amino acids, mitochondrial biogenesis, and healthspan: an evolutionary perspective. Aging (Albany NY).

[18] Goffredo M, Santoro N, Trico D, Giannini C, D'Adamo E, Zhao H, Peng G, Yu X, Lam TT, Pierpont B, Caprio S, Herzog RI (2017). A branched-chain amino acid-related metabolic signature characterizes obese adolescents with non-alcoholic fatty liver disease. Nutrients.

[19] Newgard CB (2012). Interplay between lipids and branched-chain amino acids in development of insulin resistance. Cell Metab.

[20] Batch BC, Shah SH, Newgard CB, Turer CB, Haynes C, Bain JR, Muehlbauer M, Patel MJ, Stevens RD, Appel LJ, Newby LK, Svetkey LP (2013). Branched chain amino acids are novel biomarkers for discrimination of metabolic wellness. Metabolism.

[21] Yang RY, Wang SM, Sun L, Liu JM, Li HX, Sui XF, Wang M, Xiu HL, Wang S, He Q, Dong J, Chen WX (2015). Association of branched-chain amino acids with coronary artery disease: a matched-pair case-control study. Nutr Metab Cardiovasc Dis.

[22] Yang R, Dong J, Zhao H, Li H, Guo H, Wang S, Zhang C, Wang M, Yu S, Chen W (2014). Association of branched-chain amino acids with carotid intima-media thickness and coronary artery disease risk factors. PLoS One.

[23] Menni C, Zhai G, Macgregor A, Prehn C, Romisch-Margl W, Suhre K, Adamski J, Cassidy A, Illig T, Spector TD, Valdes AM (2013). Targeted metabolomics profiles are strongly correlated with nutritional patterns in women. Metabolomics.

[24] Zhang Y, Guo K, LeBlanc RE, Loh D, Schwartz GJ, Yu YH (2007). Increasing dietary leucine intake reduces diet-induced obesity and improves glucose and cholesterol metabolism in mice via multimechanisms. Diabetes.

[25] Bifari F, Nisoli E (2017). Branched-chain amino acids differently modulate catabolic and anabolic states in mammals: a pharmacological point of view. Br J Pharmacol.

[26] Brahm A, Hegele RA (2013). Hypertriglyceridemia. Nutrients.

[27] Waterlow JC, Golden MH, Garlick PJ (1978). Protein turnover in man measured with 15N: comparison of end products and dose regimes. Am J Physiol.

[28] Leibowitz G, Cerasi E, Ketzinel-Gilad M (2008). The role of mTOR in the adaptation and failure of beta-cells in type 2 diabetes. Diabetes Obes Metab.

[29] Corsetti G, D'Antona G, Ruocco C, Stacchiotti A, Romano C, Tedesco L, Dioguardi F, Rezzani R, Nisoli E (2014). Dietary supplementation with essential amino acids boosts the beneficial effects of rosuvastatin on mouse kidney. Amino Acids.

[30] Solerte SB, Fioravanti M, Locatelli E, Bonacasa R, Zamboni M, Basso C, Mazzoleni A, Mansi V, Geroutis N, Gazzaruso C (2008). Improvement of blood glucose control and insulin sensitivity during a long-term (60 weeks) randomized study with amino acid dietary supplements in elderly subjects with type 2 diabetes mellitus. Am J Cardiol.

[31] Sun L, Hu CY, Shi XH, Zheng CG, Huang ZZ, Lv ZP, Huang J, Wan G, Qi KY, Liang SY, Zhou L, Yang Z (2013). Trans-ethnical shift of the risk genotype in the CETP I405V with longevity: a Chinese case-control study and meta-analysis. PLoS One.

[32] Sun L, Yang Z, Jin F, Zhu XQ, Qu YC, Shi XH, Wang L (2006). The Gly482Ser variant of the PPARGC1 gene is associated with Type 2 diabetes mellitus in northern Chinese, especially men. Diabet Med.

[33] Yang R, Dong J, Guo H, Li H, Wang S, Zhao H, Zhou W, Yu S, Wang M, Chen W (2013). Rapid and precise measurement of serum branched-chain and aromatic amino acids by isotope dilution liquid chromatography tandem mass spectrometry. PLoS One.

[34] Group CMCR (2004). Suggestions of Chinese Diabetes Society (CDS) for the metabolic syndrome. Chin J Diabet.

[35] Johansen D, Gronbaek M, Overvad K, Schnohr P, Andersen PK (2005). Generalized additive models applied to analysis of the relation between amount and type of alcohol and all-cause mortality. Eur J Epidemiol.

