# Supplementary material for: Association of circulating branched-chain amino acids with cardiometabolic traits differs between adults and the oldest-old
Source: Oncotarget. 2017 Oct 4;8(51):88882–93. doi: 10.18632/oncotarget.21489 (PMC5687654; doi:10.18632/oncotarget.21489)
Supplement: Supplementary file 1 [file oncotarget-08-88882-s001.pdf]

Association of circulating branched-chain amino acids with cardiometabolic traits differs between adults and the oldest-old

SUPPLEMENTARY MATERIALS

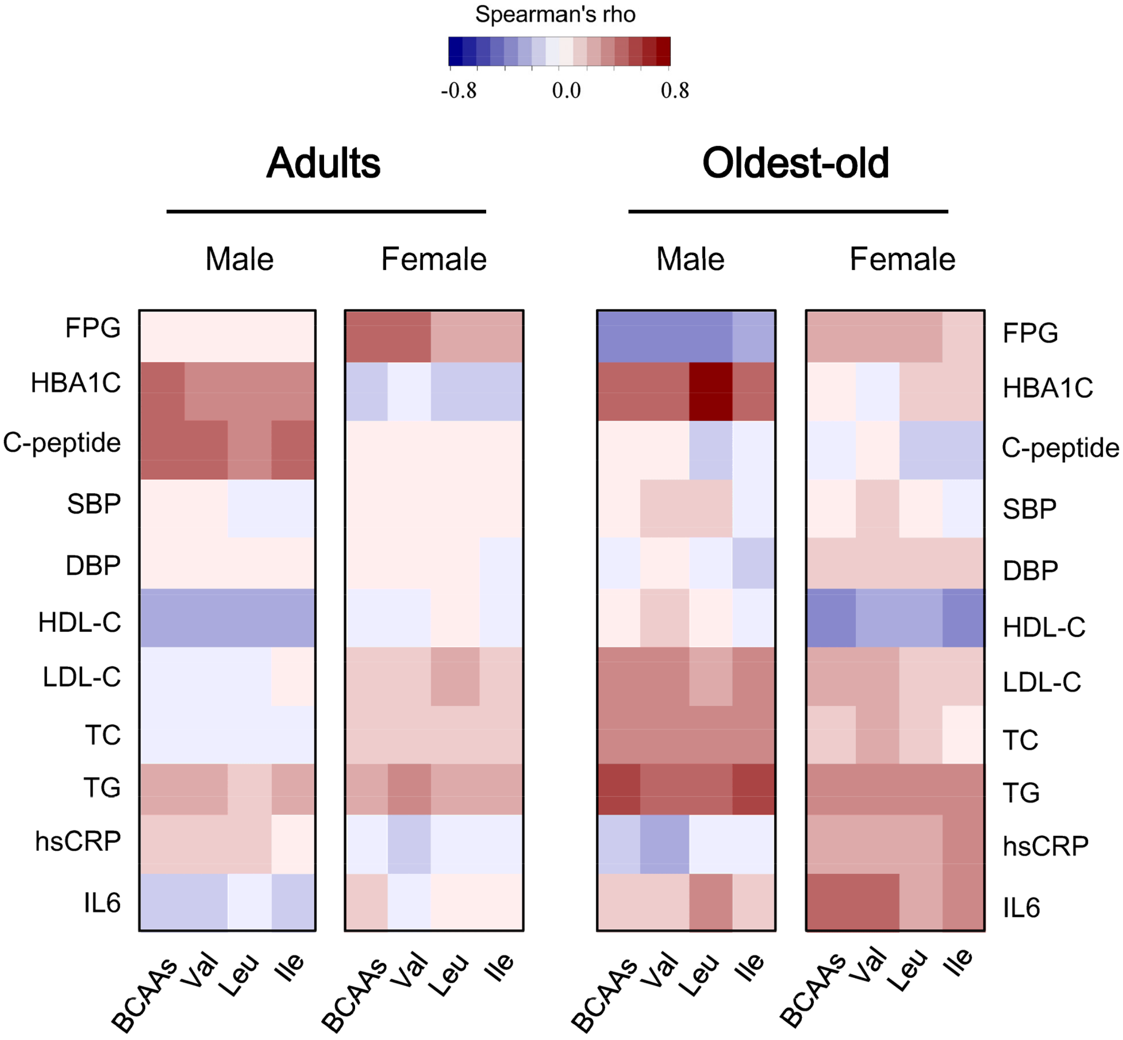

Supplementary Figure 1: Correlation heatmap illustrating the relationship between circulating BCAAs and cardiometabolic quantitative phenotypes stratified by genders. Spearman correlation coefficients are presented in a blue-white-red color scheme. Dark red indicates a more positive correlation, and dark blue indicates a more negative correlation; white indicates no correlation. We present the corresponding heatmaps for male and female subjects separately in adults and the oldest-old subjects,
